# Prophylaxis with melatonin for primary stabbing headache in pediatrics: a case report

**DOI:** 10.25100/cm.v49i2.3857

**Published:** 2018-09-30

**Authors:** Mauricio Bermúdez Salazar, Christian Andrés Rojas Cerón, Ronald Santiago Arana Muñoz

**Affiliations:** 1 Escuela de Medicina, Facultad de Salud,Universidad del Valle, Cali, Colombia

**Keywords:** Primary stabbing headache, Melatonin, Children, Case report, Ice-pick headache, Cefalea punzante primaria, Melatonina, Niños, Reporte de Caso, Cefalea en picahielos

## Abstract

**Introduction:**

Primary stabbing headache (or “ice pick headache”) is an alteration characterized by brief jabs (short stabs of pain, lasting ~3 seconds), which appear spontaneously, irregularly, and affecting unilaterally or bilaterally. Indomethacin has traditionally been used as the main therapeutic option. However, this drug is ineffective in a considerable percentage of patients and can generate multiple adverse effects that occur at therapeutic doses.

**Clinical case:**

A 7-year-old male patient with primary stabbing headache of mild to moderate intensity, lasting 3 to 4 seconds, without relevant history, with normal neurodevelopment, neurological examination and neuroimaging; no triggers were identified. It was started therapeutic trial with Coenzyme Q10; however, no improvement in the symptoms was identified.

**Treatment and outcomes:**

A therapeutic management was carried out with Melatonin, which led to complete remission of the symptoms; without adverse effects in the posterior follow-up months.

**Clinical and scientific relevance:**

There is little information regarding effective and safe treatments for primary stabbing headache in children. The present case identifies Melatonin as an innovative, effective and safe therapeutic alternative in the treatment of primary stabbing headache in children. This is a significant advance in the understanding of primary stabbing headache in the pediatric population.

**Conclusion:**

Melatonin may be an effective and safe therapeutic option for the treatment of primary stabbing headache in pediatric patients. It is necessary to deepen its research, in order to establish its use in a clinical practice guide.

## Resumen

**Introducción:**

La cefalea punzante primaria, es una alteración que se caracteriza por punzadas breves (∼3 segundos), que aparecen espontáneamente, de forma irregular y afectación unilateral o bilateral. Tradicionalmente se ha utilizado Indometacina como opción terapéutica principal. Sin embargo, este medicamento es inefectivo en un porcentaje considerable de pacientes y puede generar múltiples efectos adversos que se presentan a dosis terapéuticas.

**Caso clínico:**

Paciente masculino de 7 años de edad con cefalea punzante primaria de intensidad leve a moderada con una duración entre 3 y 4 segundos sin antecedentes relevantes, con neurodesarrollo, examen neurológico y de neuroimagen normales; no se identificaron desencadenantes. Se inició prueba terapéutica con Coenzima Q10, sin embargo no se identificó mejoría en los síntomas.

**Tratamiento y resultados:**

Se realizó un manejo terapéutico con Melatonina que conllevó a remisión completa de la sintomatología y sin efectos adversos en los meses posteriores de seguimiento.

**Relevancia clínica y científica:**

Existe poca información respecto a tratamientos efectivos y seguros para cefalea punzante primaria en niños. El presente caso identifica la Melatonina como una alternativa terapéutica innovadora, efectiva y segura en el tratamiento de la cefalea punzante primaria en niños. Lo anterior constituye un avance significativo en la comprensión de la cefalea punzante primaria en la población pediátrica.

**Conclusión:**

La melatonina puede ser una opción terapéutica efectiva y segura para el tratamiento de la cefalea punzante primaria en pacientes pediátricos. Se requiere ahondar en su investigación para establecer su uso en una guía de práctica clínica.

## Introduction

Primary stabbing headache (PSH), also known as ice pick headache or idiopathic stabbing headache, is an alteration characterized by short duration transient stabbings that appear spontaneously, in the absence of an underlying organic pathology. It is classified within the group of "other headaches," in the subgroup of "epicranial headaches" of the ICHD-3β ^1^. The stitches are repeated irregularly, anywhere on the head, unilaterally or bilaterally; sometimes, it is associated with dizziness, vomiting, photophobia and phonophobia, but without autonomic symptoms ^2^. Its estimated prevalence in children is between 3.0% - to 5.1% ^3^ ^-^ ^6^. Its diagnosis is complex because it is clinical, that is, there is no laboratory test or neuroimaging to confirm it. The pathogenesis is not well understood, and there is little information regarding effective treatments in the pediatric population ^3^. Indomethacin is considered the first-line therapeutic option for PSH; however, a considerable percentage of patients do not respond to this medication and present adverse reactions at therapeutic doses ^2^. Other alternatives for treatment include non-steroidal analgesics, acetaminophen, gabapentin, botulinum toxin type A and coenzyme Q10 ^2^ ^,^ ^3^. Clinical trials and some case reports have evidenced the effective use of Melatonin in headaches difficult to manage both in the pediatric population and in adults ^7^ ^-^ ^11^; and although little is known about the pharmacodynamics of this drug in PSH, it is believed that its possible mechanisms of action are related to its capacity to potentiate the analgesic effect of the GABAergic system and endogenous opioids, neurovascular regulation and modulation of serotonin and endorphins ^12^.

This article reports the case of a child who meets the criteria for PSH with inadequate response to conventional therapy, and whose therapeutic management with Melatonin presented favorable results ^13^.

## Case report

A 7-year-old male patient who presented at the Neurology service for a headache in his right temporal region that had evolved during one week, of intermittent nature, mild to moderate intensity, each episode between 3-4 seconds; and with subsequent, complete recovery. No history of trauma; he denies presenting fever or other systemic manifestations. There was no distal coldness, crying, or pallor, no feeling of dizziness, nausea, emesis, photophobia, phonophobia, or autonomic symptoms, no relationship with valsalva, or associated with exercise. Neither the patient nor his parents identified any triggering factors. 

The patient reports four episodes of PSH in the week prior to the consultation, without interfering with his sleep hours. There is no history of other neurological symptoms, epileptic seizures or changes in personality. The interrogation did not recognize alterations in neurodevelopment, he presents a good school performance and an adequate sleep pattern. No personal background of relevance. No headaches or other related pathologies are reported in the family history.

On physical examination, he presented normal vital signs, a weight of 22 kg and 1.22 m of height. In the neurological examination, neither alterations nor skin stigmas indicative of neuro-cutaneous syndromes were identified.

The intervention was started with a one-month observation period, in which the child's caregiver was asked to prepare a headache diary that would allow characterizing and quantifying the episodes (date and time, laterality, associated activity, duration and need for medication).

The following month, he was cited for a control, where 21 episodes were quantified with the clinical characteristics described above but without a predominant laterality; no triggering or attenuating factors were identified. A simple and contrasted cerebral magnetic resonance imaging was performed, which showed no alterations of any kind. Based on the characteristics of the episodes, the clinical course and the results of the MRI, he was diagnosed with PSH, so a therapeutic test was prescribed with Coenzyme Q10, in a dose of one 100-mg tablet given orally every 12 hours as a prophylactic treatment, and the diary registry of headaches continued.

**Figure 1:**
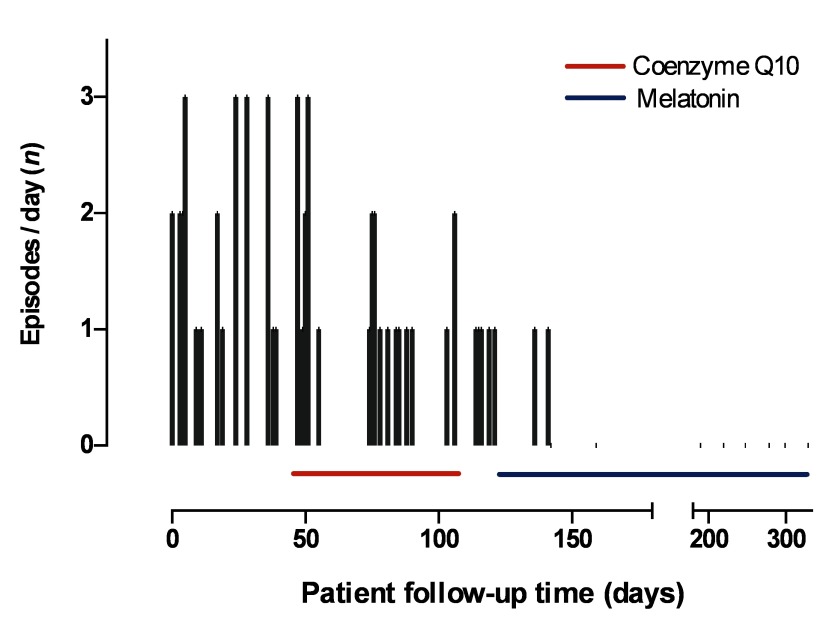
Relevant events of the case, number and frequency of episodes of primary stabbing headache.

After two months of administration of Coenzyme Q10, no improvement in symptoms was identified. Due to this therapeutic failure, it was decided to suspend that medication. Two weeks later, it was initiated a new treatment with half a tablet of 1.5 mg melatonin daily (0.07 mg/kg), administered at night; with this therapy, it was achieved a reduction in the frequency of headaches during the first two weeks of treatment: only two episodes occurred during this period. Along the six months of follow-up, no new episodes or adverse effects have been documented (Fig. 1), and both tolerability and therapeutic adherence have been optimal, as assessed by the scale of 8-item therapeutic adherence of Morisky ^14^. 

## Informed consent

Written informed consent was obtained from the patient's mother for the publication of this case report.

## Discussion

PSH was described in 1980 as "ice pick pains" of short duration (3 seconds in 80% of the occasions, rarely exceeding 2 minutes) generally unilateral, in the orbital or temporal region ^1^ ^,^ ^2^. In children, unlike what is observed in adults, there is no predominance of sex in terms of prevalence, and the average age of onset is 7 years ^4^ ^-^ ^6^.

The frequency of episodes is described as "an erratic and unpredictable alteration between symptomatic and non-symptomatic periods" ^16^, in which they can be presented from multiple episodes per day in symptomatic periods, to one per week or per year in the non-symptomatic periods ^17^. Pediatric studies report that 21-50% of patients present more than one jab (short stab of pain) per week ^4^ ^-^ ^6^. Regarding the severity of pain in pediatric patients, most studies show mild to moderate severity ^4^ ^-^ ^6^. The above agrees with the present case, in which there were identified an average of 10 episodes per month of sudden, unpredictable characteristics, of maximum 4 seconds in duration, and with a mild to moderate intensity; these episodes were intertwined with asymptomatic intervals.

PSH can occur both unilaterally and bilaterally; and the location can change in each event, and even vary in position during the same episode. Several studies show that PSH with occipital/nuchal localization occurs between 39% and 58% of cases; fronto-temporal region, between 39% and 62%; and parietal localization, 18% to 49% of cases ^3^ ^,^ ^15^ ^,^ ^16^. Usually, episodes occur spontaneously, although some triggers have been reported, such as climate changes, bright lights, lack of sleep, emotional stress and drugs ^3^ ^,^ ^15^ ^,^ ^17^ ^,^ ^18^. Comorbidities are not frequent in children, and neuroimaging studies are usually normal ^19^. In the current case, the episodes were unilateral and mostly fronto-temporal, but without predominant laterality; no triggers or comorbidities were identified, and the neuroimaging study was normal.

The pathogenesis of PSH is not very well understood; some theories range from disorders in the peripheral branches of the trigeminal nerve to dysfunctions in the central mechanisms of pain control ^3^ ^,^ ^16^.

The treatment of PSH poses a therapeutic challenge because of the sudden, brief (in the order of seconds) and unexpected onset of headache, which prevents the establishment of analgesics for acute treatment; therefore, prophylactic therapy constitutes the cornerstone of management, and its objective is to reduce or eradicate the episodes of headache. This therapy involves continuous and prolonged exposure to a drug, with the potential risk of manifestation of adverse effects.

Indomethacin has traditionally been used as the main therapeutic option for PSH in adults and children. However, its prolonged use can generate adverse reactions, such as: dyspepsia, abdominal pain, gastric ulcers, renal failure, dizziness, vomiting, anorexia, diarrhea, fatigue, confusion, depression and psychosis. There have also been reported cases of fatal liver involvement, acute pancreatitis and aplastic anemia ^2^ ^,^ ^20^. It has been reported that 35% of patients with PSH do not respond to indomethacin, and that more than 35% have adverse effects at therapeutic doses ^2^ ^,^ ^20^.

In addition, the prolonged use of non-steroidal analgesics and acetaminophen has been associated with an increased risk of hepatotoxicity, nephrotoxicity ^21^, and headache due to overuse of analgesics, when the latter are used for 15 days or more per month in a period greater than 3 months ^1^ ^,^ ^22^ ^,^ ^23^.

Due to the above, other pharmacological options were considered; for example, improvement has been documented in adult patients treated with Gabapentin and botulinum toxin type A ^2^ ^,^ ^3^. The use of botulinum toxin was not considered for the present case because it is an invasive and more expensive treatment, nor was the use of gabapentin because of the risk of alterations in behavior and learning ^24^. Another alternative is Coenzyme Q10, which exhibits anti-inflammatory and antioxidant properties and rarely presents adverse effects ^25^ ^,^ ^26^; however, its use was not effective in this patient.

It was reported that the use of 3-12 mg of melatonin at night achieved a complete remission in adult patients with PSH, who had been initially administered indomethacin without success ^11^. However, there were not found clinical trials comparing the therapeutic effectiveness of Indomethacin with Melatonin.

Effective therapeutic use of melatonin in a range of 0.3-10 mg has been reported in different pediatric conditions ^9^; for example, randomized trials in children with migraine or tension-type headache demonstrated a favorable response to treatment with 3 mg of melatonin ^8^ ^-^ ^11^. Additionally, there have been reported cases of children with cluster headache and hypnic headache treated with 4-10 mg of melatonin, who reached optimal results ^8^. However, no reports of melatonin use were found in pediatric patients with PSH.

Melatonin is a pineal hormone with anti-inflammatory, analgesic, antioxidant and anxiolytic properties; it is structurally similar to indomethacin, but with fewer adverse effects ^2^ ^,^ ^8^ ^-^ ^11^. It has been used in different types of primary headache, although its mechanism of action in PSH is unknown ^8^. However, some of its properties that could be related to its therapeutic result in PSH include:

1. its anti-inflammatory effect by preventing the translocation and binding of NF-kB with DNA and inhibition of the production of adhesion molecules necessary for diapedesis;
2. its ability to directly eliminate toxic free radicals;
3. antagonism of glutamate release avoiding neurotoxicity;
4. potentiation of the neurotransmission of the GABAergic system and of the analgesic efficacy of endogenous opioids, since it behaves as an opioid receptor agonist;
5. it also participates in cerebrovascular regulation by increasing the vasoconstrictor effect of norepinephrine and modulating the neurotransmission of serotonin and endorphins ^12^.

Multiple studies have evaluated its safety; for example, the administration of 10 mg/kg of intravenous melatonin has been reported in neonates with procedural pain, and 700 mg in patients with metastatic melanoma, without toxicity in any case; nor significant adverse effects ^8^ ^-^ ^11^. Studies indicate, as possible adverse effects, agitation, dizziness, nausea, drowsiness and headache, all of mild intensity; however, the distribution of these effects does not differ in frequency with those of the placebo group ^8^ ^-^ ^11^. A lethal dose (LD_50_) has not been estimated yet, because the upper limit of drug solubility was reached without toxicity ^8^ ^-^ ^11^.

In the present case, Melatonin was initiated at low doses, in order to titrate the medication according to the therapeutic response and the presence of adverse effects. However, the use of 1.5 mg of melatonin per day, orally and administered at night, showed a rapid decrease in the frequency of headache episodes, shortly after the treatment was established, until they completely disappeared; and no adverse effects associated with its use during follow-up were identified.

## Conclusion

Melatonin may be an effective and safe therapeutic option for the treatment of PSH in pediatric patients, compared to the conventional treatment. It is necessary to evaluate the dose and the long-term therapeutic effect.
