# Supplementary material for: Prophylaxis with melatonin for primary stabbing headache in pediatrics: a case report
Source: Colomb Med (Cali). 2018 Sep 30;49(3):244–8. doi: 10.25100/cm.v49i2.3857 (PMC6220483; doi:10.25100/cm.v49i2.3857)
Supplement: Related texts

# Case report: Profilaxis con melatonina para cefalea punzante primaria en pediatría: reporte de caso

## Introducción 

La cefalea punzante primaria (CPP), también conocida como cefalea en picahielos o punzante idiopática, es una alteración caracterizada por punzadas transitorias de corta duración que aparecen espontáneamente en ausencia de una patología orgánica de base. Se clasifica dentro del grupo de “otras cefaleas” y el subgrupo de “cefaleas epicraneales” de la ICHD-3β ^1^. Las punzadas se repiten de forma irregular, en cualquier lugar de la cabeza, unilateral o bilateralmente; en ocasiones se asocia con mareo, vómito, fotofobia y fonofobia pero sin síntomas autonómicos ^2^. Su prevalencia estimada en niños esta entre el 3.0%- al 5.1% ^3^^-^^6^. Su diagnóstico es complejo debido a que es clínico, es decir, no existe una prueba de laboratorio o neuroimagen que lo confirmen. La patogénesis no está bien dilucidada y hay poca información respecto a tratamientos efectivos en la población pediátrica ^3^. La Indometacina es considerada la opción terapéutica de primera línea para la CPP; no obstante, un porcentaje considerable de pacientes no responden a este medicamento y presentan reacciones adversas a las dosis terapéuticas ^2^. Otras alternativas para el tratamiento incluyen los analgésicos no esteroideos, el Acetaminofén, la Gabapentina, la Toxina botulínica tipo A y la Coenzima Q10 ^2^^,^^3^. Ensayos clínicos y algunos reportes de caso evidenciaron el uso efectivo de la Melatonina en cefaleas de difícil manejo tanto en la población pediátrica como en los adultos ^7^^-^^11^, y, aunque se sabe poco acerca de la farmacodinamia de este medicamento en la CPP, se cree que sus posibles mecanismos de acción se relacionan con sus capacidades de potenciación del efecto analgésico del sistema gabaérgico y los opioides endógenos, regulación neurovascular y modulación de la serotonina y las endorfinas ^12^. 

El presente artículo reporta el caso de un niño que cumple con los criterios para CPP con respuesta inadecuada a la terapia convencional y cuyo manejo terapéutico con Melatonina presentó resultados favorables ^13^.

## Reporte de caso

Paciente masculino de 7 años quien se presentó en el servicio de Neurología por dolor de cabeza en la región temporal derecha de una semana de evolución, de carácter intermitente, intensidad leve a moderada, con una duración de entre 3-4 segundos y con posterior recuperación completa. No presentó antecedentes de trauma, niega fiebre y otras manifestaciones sistémicas. No presentó frialdad distal, llanto, ni palidez, tampoco se encontró una sensación de mareo, náuseas, emesis, fotofobia, fonofobia, ni síntomas autonómicos, no se relacionó con valsalva ni se asoció al ejercicio. El paciente y los padres no identificaron factores desencadenantes. 

El paciente refiere cuatro episodios de CCP en la semana previa a la consulta, sin que interfieran con sus horas de sueño. No hay historia de otros síntomas neurológicos, crisis epilépticas ni cambios en la personalidad. Al interrogatorio no se reconocieron alteraciones en el neurodesarrollo, tiene un buen rendimiento escolar y un patrón adecuado de sueño. Sin antecedentes personales de relevancia. En la historia familiar no se reportan casos de cefalea u otra patología relacionada. 

Al examen físico, presentó signos vitales normales, un peso de 22 kg y 1.22 m de estatura. En el examen neurológico no se identificaron alteraciones ni tampoco estigmas en piel indicativos de síndromes neurocutáneos. 

Se comenzó la intervención con un periodo de observación de un mes en el cual se pidió al acudiente del niño la elaboración de un diario de cefalea que permitiera caracterizar y cuantificar los episodios (fecha y hora, lateralidad, actividad asociada, duración y necesidad de medicación). 

Al mes siguiente se citó a un control, donde se cuantificaron 21 episodios con las características clínicas descritas anteriormente pero sin una lateralidad predominante, tampoco se identificaron factores desencadenantes ni atenuantes. Se realizó una resonancia nuclear magnética cerebral simple y contrastada, que no demostró alteraciones de ningún tipo. Con base en las características de los episodios, el curso clínico y los resultados de la resonancia magnética, se diagnosticó CPP, por lo cual se prescribió una prueba terapéutica con Coenzima Q10, en una dosis de una tableta de 100 mg vía oral cada 12 horas como tratamiento profiláctico, y se continuó con el diario de cefaleas. 

Luego de dos meses de administración de la Coenzima Q10 no se identificó ninguna mejoría en los síntomas; debido a este fracaso terapéutico se decidió suspender dicha medicación. Dos semanas más tarde se inició un tratamiento con media tableta de melatonina, 1.5 mg diarios vía oral (0.07 mg/kg) administrados en la noche; con esta terapia se identificó una reducción de la frecuencia de las cefaleas durante las primeras dos semanas de uso, lapso en el cual se presentaron dos episodios. Durante los seis meses de seguimiento posteriores no se han documentado nuevos episodios ni efectos adversos (Fig. 1), y tanto la tolerabilidad como la adherencia terapéutica han sido óptimas según lo evaluado mediante la escala de adherencia terapéutica de 8 ítems de Morisky ^14^.

**Figura. 1:** **Figura 1.** Acontecimientos relevantes del caso, cantidad y frecuencia de los episodios de cefalea punzante primaria.

### Consentimiento informado

Se obtuvo el consentimiento informado por escrito de la madre del paciente para la publicación de este reporte de caso. 

## Discusión

La CPP fue descrita en 1980 como un dolor en “picahielos” de corta duración (3 segundos en el 80% de las ocasiones, rara vez sobrepasa los 2 minutos) generalmente unilateral en la región orbital o temporal ^1^^,^^2^. En niños, a diferencia de lo observado en adultos, no hay una predominancia del sexo en cuanto a la prevalencia, y la edad promedio de aparición es a los 7 años ^4^^-^^6^.

La frecuencia de los episodios se describen como “una alteración errática e impredecible entre periodos sintomáticos y no sintomáticos” ^16^, en la cual se pueden presentar desde múltiples episodios por día en los periodos sintomáticos, a uno por semana o por año en los periodos no sintomáticos ^17^. Los estudios pediátricos reportan que del 21-50% de los pacientes presentan más de una punzada por semana ^4^^-^^6^. Con respecto a la severidad del dolor en pacientes pediátricos, la mayoría de estudios muestran una gravedad de leve a moderada ^4^^-^^6^. Lo anterior concuerda con el presente caso, en el que se identificó un promedio de 10 episodios mensuales de características súbita e impredecible, de máximo 4 segundos de duración, con una intensidad de leve a moderada y que se alternaron con intervalos asintomáticos. 

La CPP puede ocurrir tanto unilateral como bilateralmente y cambiar la localización en cada evento, e incluso variar de posición durante un mismo episodio. Diversos estudios muestran que la CPP con localización occipital/nucal ocurre entre el 39% y el 58% de los casos, en la región fronto-temporal entre el 39% y el 62% y tiene localización parietal en el 18% al 49% ^3^^,^^15^^,^^16^. Usualmente los episodios ocurren de manera espontánea, aunque se han reportado algunos desencadenantes como cambios climáticos, luces brillantes, falta de sueño, estrés emocional y fármacos ^3^^,^^15^^,^^17^^,^^18^. Las comorbilidades no son frecuentes en niños y los estudios de neuroimagen suelen ser normales ^19^. En el caso actual los episodios fueron unilaterales y en su mayoría con ubicación fronto-temporal pero sin una lateralidad predominante, no se identificaron desencadenantes, ni comorbilidades y el estudio por neuroimagen fue normal. 

La patogénesis de la CPP no está muy bien dilucidada, algunas teorías plantean desde trastornos en las ramas periféricas del nervio trigémino hasta disfunciones en los mecanismos centrales de control del dolor ^3^^,^^16^.

El tratamiento de la CPP plantea un reto terapéutico, debido a que la aparición súbita, breve (del orden de segundos) e inesperada de la cefalea, impide la instauración de analgésicos para el tratamiento agudo, por lo tanto la terapia profiláctica constituye la piedra angular del manejo y su objetivo es disminuir o erradicar los episodios de cefalea. Esta terapia, implica la exposición continua y prolongada a un fármaco con el riesgo potencial de manifestación de efectos adversos. 

Tradicionalmente se ha utilizado Indometacina como opción terapéutica principal para la CPP en adultos y en niños. Sin embargo, su uso prolongado puede generar reacciones adversas como: dispepsia, dolor abdominal, ulceras gástricas, falla renal, mareo, vomito, anorexia, diarrea, fatiga, confusión, depresión y psicosis, también se han reportado casos de compromiso hepático fatal, pancreatitis aguda y anemia aplásica ^2^^,^^20^. Se ha reportado que el 35% de los pacientes con CPP no responden a la Indometacina y que más del 35% presentan efectos adversos a dosis terapéuticas ^2^^,^^20^. 

Además, el uso prolongado de analgésicos no esteroideos y acetaminofén se ha asociado con un mayor riesgo de hepatotoxicidad, nefrotoxicidad ^**(21)**^ y cefalea por sobreuso de analgésicos cuando estos últimos se utilizan por 15 días o más al mes en un periodo mayor a 3 meses ^1^^,^^22^^,^^23^. 

Debido a lo anterior se consideraron otras opciones farmacológicas, por ejemplo, se ha documentado mejoría en pacientes adultos tratados con Gabapentina y Toxina botulínica tipo A ^2^^,^^3^. No se consideró el uso de Toxina botulínica para el presente caso por tratarse de un tratamiento invasivo y de mayor costo, tampoco el uso de Gabapentina debido al riesgo de alteraciones en la conducta y el aprendizaje ^24^. Otra alternativa es la Coenzima Q10, que exhibe propiedades antiinflamatorias y antioxidantes y rara vez presenta efectos adversos ^25^^,^^26^, no obstante, su uso no fue efectivo en el paciente.

Se reportó que el uso de 3-12 mg de melatonina en las noches logró una remisión completa en pacientes adultos con CPP a quienes se les había administrado inicialmente Indometacina sin éxito ^11^. No obstante, no se encontraron ensayos clínicos que comparen a la efectividad terapéutica de la Indometacina con la Melatonina. 

Se ha reportado el uso terapéutico efectivo de melatonina en un rango de 0.3-10 mg en diferentes condiciones pediátricas ^9^; por ejemplo, ensayos aleatorizados realizados en niños con migraña o cefalea tensional demostraron una respuesta favorable al tratamiento con 3 mg de melatonina ^8^^-^^11^. Adicionalmente se reportan casos de niños con cefalea en racimos y cefalea hípnica tratados con 4-10 mg de melatonina que alcanzaron resultados óptimos ^8^. Sin embargo, no se encontraron reportes del uso de melatonina en pacientes pediátricos con CPP. 

La Melatonina es una hormona pineal con propiedades antiinflamatorias, analgésicas, antioxidantes y ansiolíticas; es estructuralmente similar a la Indometacina pero presenta menos efectos adversos ^2^^,^^8^^-^^11^. Ha sido utilizada en diferentes tipos de cefalea primaria, aunque su mecanismo de acción en la CPP se desconoce ^8^. Sin embargo, algunos de sus propiedades que podrían relacionarse con su resultado terapéutico en la CPP incluyen:

1. su efecto antiinflamatorio al prevenir la translocación y enlace del NF-kB con el ADN e inhibir la producción de moléculas de adhesión necesarias para la diapédesis;
2. su habilidad para eliminar directamente radicales libres tóxicos;
3. antagonismo de la liberación de glutamato evitando la neurotoxicidad;
4. potenciación de la neurotransmisión del sistema gabaérgico y de la eficacia analgésica de los opioides endógenos ya que se comporta como un agonista de los receptores opioides;
5. también participa en la regulación cerebrovascular al aumentar el efecto vasoconstrictor de la norepinefrina y modular la neurotransmisión de la serotonina y las endorfinas ^12^.

Múltiples estudios han evaluado su seguridad, por ejemplo, se ha reportado la administración de 10 mg/kg de melatonina intravenosa en neonatos con dolor procedimental y de 700 mg en pacientes con melanoma metastásico sin toxicidad en ambos casos, ni efectos adversos significativos ^8^^-^^11^. Estudios indican como posibles efectos adversos agitación, mareo, náuseas, somnolencia y dolor de cabeza, todos de intensidad leve; sin embargo, la distribución de estos efectos no difiere en frecuencia con los del grupo placebo ^8^^-^^11^. Tampoco se ha estimado una dosis letal 50 debido a que se alcanzó el límite superior de solubilidad del medicamento sin que se presentara toxicidad ^8^^-^^11^.

En el presente caso la Melatonina fue iniciada a dosis bajas con el objetivo de titular la medicación según la respuesta terapéutica y la presencia de efectos adversos. No obstante, el uso de 1.5 mg de melatonina por día vía oral y administrados en la noche, mostró, al poco tiempo de instaurado el tratamiento, una rápida disminución en la frecuencia de los episodios de cefalea hasta alcanzar su desaparición completa y no se identificó ningún efecto adverso asociado a su uso durante el seguimiento.

## Conclusión

La melatonina puede ser una opción terapéutica efectiva y segura para el tratamiento de la CPP en pacientes pediátricos en comparación con el tratamiento convencional. Se requiere evaluar la dosis y el efecto terapéutico a largo plazo.
